# Applying a Power Analysis to Everything We Do: A Qualitative Inquiry to Decolonize the Global Health and Development Project Cycle

**DOI:** 10.9745/GHSP-D-23-00187

**Published:** 2023-10-30

**Authors:** Doreen Tuhebwe, Sarah Brittingham, Amandari Kanagaratnam, Elikem Togo, Funmilola M. OlaOlorun, Rhoda K. Wanyenze, Ndola Prata, Allysha C. Maragh-Bass

**Affiliations:** a Makerere University School of Public Health, Kampala, Uganda.; b FHI 360, Durham, NC, USA.; c The George Washington University Milken Institute School of Public Health, Washington, DC, USA.; d Evidence for Sustainable Human Development Systems in Africa; Department of Community Medicine, College of Medicine, University of Ibadan, Ibadan, Nigeria.; e Evidence for Sustainable Human Development Systems in Africa; School of Public Health, University of California, Berkeley, CA, USA.

## Abstract

**Background:**

Global health and development (GHD) systems that centralize power in the Global North were conceived during colonialism. As a result, they often replicate unequal power structures, maintaining dogged inequities. Growing and historic calls to decolonize GHD advocate for the transfer of power to actors in the Global South. This article identifies examples of colonial legacies in today's GHD projects and offers actionable strategies to decolonize.

**Methods:**

From August 2021 to March 2022, 20 key informants across 15 organizations participated in interviews about their experiences and perspectives relating to the decolonization of GHD. We used deductive thematic coding to identify examples of challenges and strategies to address them across 3 project life cycle phases: conceptualization and contracting, program planning and implementation, and program evaluation and dissemination.

**Results:**

Participants described how power is maintained in the Global North, sharing countless examples across the project life cycle, including agenda-setting with minimal local participation or partnership, onerous requirements that limit grantee eligibility, Global North ownership of data collected by and in the Global South, and dissemination in languages and formats that are not easily accessible to Global South audiences. Proposed strategies to decolonize GHD projects include having built-in participatory processes and accountability mechanisms; aligning solicitations with existing local strategies; adapting the process for awarding, contracting, and evaluating investments to increase the representation and competitiveness of Global South entities; creating trusting, respectful relationships with Global South partners; and systematically applying power analyses to each step of the project life cycle.

**Conclusions:**

GHD practitioners suggested project life cycle-based strategies for shifting power and redistributing resources, which we argue will ultimately enhance the value, impact, and sustainability of GHD programming.

## Abstract (precis)

This study contributes evidence characterizing the legacy of colonialism in global health and development and reinforces calls to recenter Global South expertise and leadership from the perspectives of experienced practitioners in the Global South and North.

## Teaser

**KEY MESSAGES:**

- Colonial legacies that maintain power and control in the Global North are present throughout the project life cycle phases of conceptualization and contracting, planning and implementation, and evaluation and dissemination.
- Using qualitative methodologies, we interrogate this legacy to identify concrete strategies for funders, implementers, and all partners in global health and development to decolonize their work. The key findings and recommendations from this study, which are well-poised for wide dissemination, have tangible action steps and grounding in lived experiences, which, if taken, can lead to a more just and equitable field.

**Key Implications:**

- For funders, experienced practitioners offer several strategies that can be implemented to better align solicitations to local context, support nontraditional awardees to apply, embrace more flexibility in implementation, and utilize more nuanced metrics to evaluate investments.
- For implementers based in the Global North and the Global South, suggestions emerged to move the needle toward restoring power to people, organizations, and communities in the Global South by building in more accountability, redistributing resources, increasing representation, and disseminating results in ways that are most likely to reach those who can use them.

## INTRODUCTION

Global health and development (GHD) is encumbered by colonial legacies that trace back to its origins.^1^^–^^5^ It is a vast enterprise that, in many iterations, continues to privilege the Global North (GN) over the Global South (GS), “western” scientific knowledge over indigenous, and white over Black.^4^^,^^6^^–^^9^ Although we acknowledge that much of the terminology we have used in this article (Global North, Global South, development, implementer, project) to describe GHD is problematic and perpetuates colonial legacies, we elected to use this outdated language for ease of understanding. Our definitions are shared in a white paper.^10^

The growing and historic call to decolonize GHD invites us to interrogate the system and structures upon which GHD lies and advocates for the dismantling of power structures that maintain these resolute inequities.^1^^,^^5^^,^^11^ Movements to decolonize have garnered diverse reactions from GHD practitioners, notwithstanding the question as to whether GHD will “survive its decolonization.”^2^ In the white paper, we make the case for decolonizing GHD and explore the relationship of decolonization with 2 interrelated strategies to shift power: (1) diversity, equity, inclusion, and accessibility and (2) localization. The white paper also addresses the problematic lexicon of GHD.^12^ To date, the literature offers few concrete approaches to address the underlying power asymmetry that hinders GHD implementation.^13^^,^^14^ The legacies of colonialism have been well documented by various scholars and international organizations,^15^^–^^18^ but the perspectives of those involved in implementing GHD projects are less well documented. We believe that in contexts where most GHD work is grant funded through official development assistance by donor country governments and private foundations or corporations,^19^ a project life cycle lens offers an important vantage point from which to identify and address the colonial legacies at each phase of a GHD project. The expanded project life cycle that we adopted includes pre-award and post-award processes^20^ and highlights conceptualization, contracting, planning, implementation, evaluation, and dissemination as key processes whereby these legacies impede GHD, necessitating strategies to decolonize.^10^

A project life cycle lens offers an important vantage point from which to identify and address the colonial legacies at each phase of a GHD project.

In this article, we describe the presence and challenges of colonial legacies in grant-funded GHD projects and suggest tangible approaches to redress those legacies. Examples of colonial legacies in today's GHD project phases are reported via the voices of GHD practitioners across the GS and GN.^1^ We hope to contribute to discussion beyond grant-funded GHD projects, as these concepts apply to most GHD partnerships and collaborations. Although no set of actions can restore the dignity stolen by colonization, suggestions for operational strategies to decolonize funding and implementation processes are proposed, with specific actions that can move the needle toward restoring power to people, organizations, and communities.

## METHODS

### Study Design

As part of the Research for Scalable Solutions Consortium, which is composed of universities and international nongovernmental organizations, we purposively drew from our global network of GHD professionals to identify 20 key informants who have firsthand knowledge and depth of experience in the field of GHD. Key informant interviews were conducted with 15 individuals from the GS and 5 from the GN across 15 organizations. We developed an interview guide to elicit each person's perspectives about key themes related to decolonization. The interview guide was translated into French. The terms decolonization, diversity, equity, inclusion, and accessibility, and localization were introduced to each participant as part of the interview guide. The interview guide focused on the ongoing debates on decolonization, diversity, equity, and inclusion in global health work and programs; how the legacy of colonialism and its related power structures play out in the participants' work in GHD; ways through which the legacy of colonialism is impacting program implementation; strategies to make South-South and North-South collaborations more equitable and transparent; what the participants' organization has done to combat discrimination, increase diversity, and create an equitable environment in their leadership and workplace culture; and the definition of localization and how participants have seen localization play out in their own work.

A semistructured approach allowed informants to freely raise their unique concerns based on their relevant lived experiences.^21^^,^^22^ Depending on the informants' experiences, the interviewers probed as necessary with the aim of exhausting the 6 key themes.

### Participant Recruitment

Interviewers were situated at 3 different institutions that are part of the Research for Scalable Solutions Consortium^23^: Makerere University School of Public Health in Uganda; Evidence for Sustainable Human Development Systems in Africa, a GHD consultancy in Cameroon; and FHI 360, a large, U.S.-based, international nongovernmental organization. Key informants were purposively selected from the authors' network and identified based on existing interest in and experience with power dynamics in GHD, previous engagement in GHD research or programs, and tenure in GHD (i.e., experience in GHD). Six key informants were employed in academia as public health professionals. They often serve as principal investigators in GHD projects engaged in research, implementation, and community service. We classified the tenure and positions of each as junior-level (less than 5 years of experience), mid-level (5–10 years of experience), or senior-level (more than 10 years of experience). All participants provided informed consent. Informants were not incentivized for their time.

### Data Collection

Key informant interviews lasting between 30 to 90 minutes were conducted in English or French by trained interviewers from each of the 3 institutions via an online platform or phone call. To reduce bias, most interviewers were not previously familiar with the informants they interviewed. Before the interview, 1 interviewer was familiar with 2 key informants, and another was familiar with 1 key informant. In cases in which they were familiar, interviewers were matched with informants with similar career levels. All but 1 participant gave consent to audio-record the interviews. For the informant who did not agree to audio-recording, detailed notes were taken. All interviewers used a note-taking template to capture themes and information from interviews; afterward, these notes were checked for completeness by interviewers and supplemented with additional notes based on the recording, when available. Transcripts were used for all interviews that were audio-recorded. During interviews, the team met in real time to discuss preliminary findings; interviews were completed when theoretical saturation was reached (i.e., consistency across themes from informants, which typically occurs with 15 to 20 interviews).^24^

### Data Analysis

An analytical framework was adopted before coding that identified challenges related to colonial legacies and strategies to decolonize across 3 distinct project phases. A codebook informed by the framework with deductive codes for challenges related to legacies of colonialism and strategies to decolonize across each GHD project life cycle phase was developed and iterated throughout the analysis process (Supplement). We used an adapted version of grounded theory with constant comparison analyses.^25^ To build consensus, 3 coders coded the first 3 transcripts. After the coding of each transcript, the coders reviewed how they each applied the codes, discussing discrepancies and resolving issues in a series of consensus meetings. In the discussion of the second transcript, the coders noted that they had applied 2 codes inconsistently due to differences in how the term diversity, equity, inclusion, and accessibility is used in the GN and GS; this was resolved. In total, 3 interviews (15% of the sample) were coded by each of 3 coders to ensure we were applying the codes consistently, drawing connection between coding categories (i.e., project life cycle challenges and strategies), reconciling any discrepancies, coming to agreement, and iterating the codebook. The remaining 17 interviews were split among the coders and single coded. Coders extracted exemplar quotes from their transcripts and used ATLAS.ti 22 software to analyze all data.

Results present colonial legacies and strategies to decolonize across 3 GHD project phases: (1) conceptualization and contracting, (2) planning and implementation, and (3) evaluation and dissemination. We did not aim to compare across countries, regions, or types of respondents.

### Reflexivity

We acknowledge that our study team is mostly comprised of highly educated researchers and professors with access to many privileges, including long-term working relationships across consortia of GS-GN partnerships. At each stage of data collection, coauthors met to note key observations and discuss analytic processes and memos. These meetings continued during analyses and informed the positionality of all authors. Analyses intentionally did not attempt to reflect the views of the authors' organizations; rather, interpretation is from each author's perspective and unique lived experience, which span GS and GN.

### Ethical Approval

The study was approved by the Makerere University School of Public Health Higher Degrees Research and Ethics Committee (Protocol #816) and the National Council for Science and Technology in Uganda (Reference #HS708ES). FHI 360's Office of International Research Ethics in the United States issued a nonresearch determination, and Evidence for Sustainable Human Development Systems in Africa used an Institutional Review Board reliance agreement with FHI 360's Institutional Review Board. From development of the concept note to development of the article, this process was highly collaborative across the institutions.

## RESULTS

### Description of Participants

Table 1 outlines the types of institutions and number of key informants included in the sample. Sixteen participants worked in senior-level leadership positions (e.g., director, founder, manager), and the remaining 4 mid- and junior-level positions identified as a doctoral student, associate professor, researcher, and lecturer.

**TABLE 1.** Characteristics of Key Informants Interviewed About Power Dynamics in Global Health and Development

Type of Institution (N=15)	Participants, No. (N=20)
University^a^	6
Funding organization	4
International nongovernmental organization	3
Community-based organization^b^	3
Nongovernmental organization^c^	2
Civil society	2

a Participants from universities are public health professionals engaged in implementation who often serve as principal investigators in global health and development research and projects.

b Community-based organization represents small, community-level organizations that are subawardees reporting to national Global South partners. Community-based organizations do not have national presence.

c Nongovernmental organization represents a nonprofit organization that works with the government and very closely with the community. They are independent of government and aim to further a social or humanitarian mission. A nongovernmental organization may have national presence.

Throughout the interviews, participants noted the presence of tangible colonial legacies and offered strategies to address them. These challenges and opportunities are organized within the context of the GHD project life cycle. The definitions applied to each phase of the project life cycle are shown in the Figure. Although not an exhaustive descriptor of the numerous programs, research studies, and initiatives that are implemented, we have chosen to use the term “project” to be inclusive of the wide spectrum of GHD efforts in the GS. Table 2 summarizes key challenges and related strategies from each of the project phases.

**TABLE 2.** Summary of Key Challenges and Strategies by Project Phase

Challenges/Colonial Legacies	Strategies to Decolonize
Conceptualization and contracting phase	
Priorities set/driven by GN and misaligned to the issues/context	Align solicitations with local priorities, policies, agendas, and strategies through processes such as reverse funding calls. Develop local partnerships for awards and be accountable to targets such as funding allocations and number of lead GS-based project directors.
Burdensome contracting processes; lack of bandwidth to search for opportunities among GS	Reduce administrative burdens that present barriers to diversify applicants. For example, develop a dedicated unit to assist organizations with the paperwork and accept concepts in various formats, including videos.
Lack of negotiation power/skills Inequitable institutional investment by funders in local organizations	Form GS-based coalitions to share resources and power. Increase representation of small, locally led organizations. Invest in GS-based organizations to build systems to manage the administrative processes of applying for and receiving funds by relaxing unequal overhead limits. Define equitable partnership in GHD.
Planning and implementation phase	
Unequal distribution of resources	Allocate resources that allow GS-based organizations to build the systems needed to increase their ability to manage grant implementation. Increase GS spending and decrease GN spending.
Undervalued roles; extractive dynamic	Acknowledge and value GS roles in research, including but not limited to local expertise, shepherding of relationships with participants and other stakeholders, data collection and analysis, and more.
Lack of flexibility in implementation	Listen to the GS. Open a line of communication between donors and funded partners, especially those based in GS, to allow for more adaptive management and responsiveness to context. Build in accountability and feedback loops whereby funders and implementers are accountable to communities and subpartners.
Evaluation and dissemination phase	
Co-option of work	Facilitate relationship-building and communication between GS partners and GN funders. GN partners should champion and cede space to promote the presence and visibility of GS partners when communicating with funders. Data collected by and from the GS should be co-owned by GS partners.
GN-oriented dissemination and research utilization	When planning for dissemination and knowledge products, prioritize what will be most valuable to the GS context from which the insights were generated. Prioritize reaching end users of outputs by using local languages and accessible/relatable formats as opposed to academic manuscripts. Facilitate/make space for leadership/meaningful participation of GS colleagues in the development of manuscripts. Ensure authorship discussions do not privilege skills such as English as a first language that do not necessarily correlate to the value of contributions.
Reliance on quantitative results	Use more nuanced evaluation of funder investments.

Abbreviation: GHD, global health and development; GN, Global North; GS, Global South.

**FIGURE.**
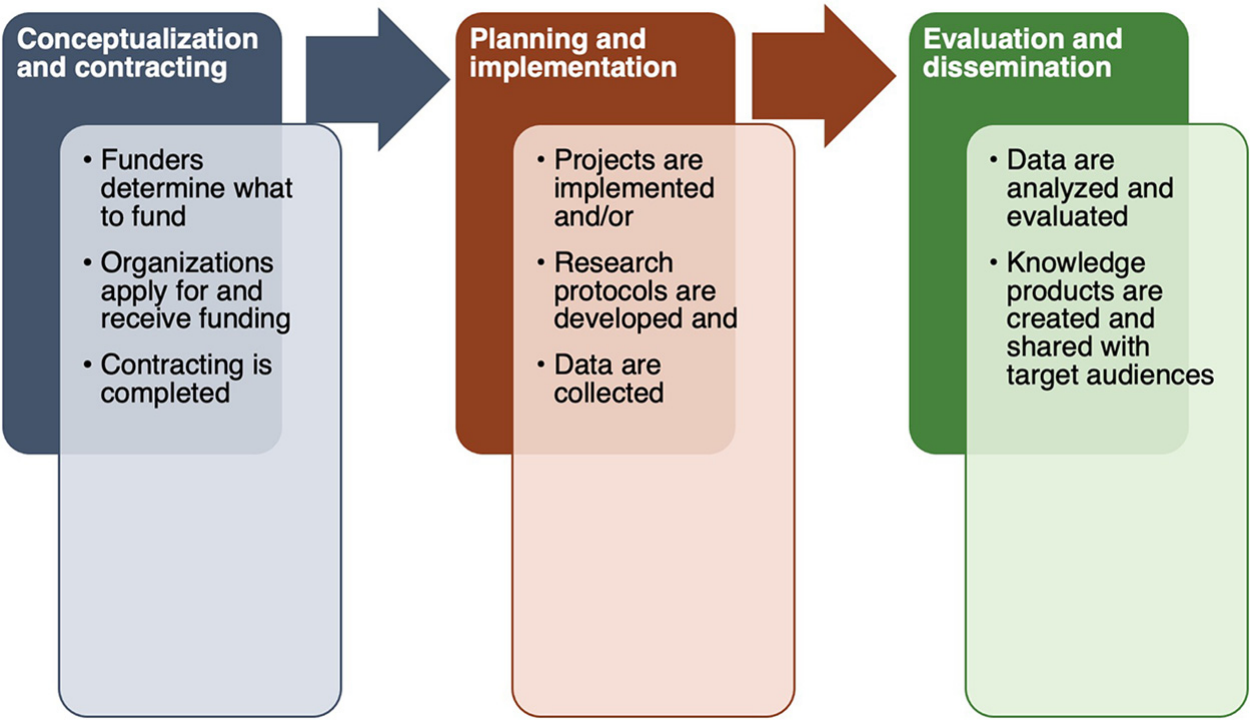
Global Health and Development Project and Grant-Making Phases and Definitions

### Colonial Legacies in Conceptualization and Contracting and Strategies to Address Them

Reflecting on the presence of challenges stemming from colonial legacies, key informants noted how the power asymmetry between GS and GN emerges during the conceptualization and contracting phases in several specific ways (Table 2).

*The funders are the ones that set the priority, you see, the funders are the ones that say we won't give unless you are partnering with this institution or what. So, you get partners and well that is tricky … Most of the funders are in the global north and they say we fund grants in this areas and this area. Sometimes those are even not relevant so you will also go ahead, I think it is a cascade …* —Junior-level, GS

A senior-level GS participant said, “but the actual problem is not being tackled head-on.”

*The way we do business, we put out solicitations that are based on what we think is the way to achieve something. Those get awarded to the people who are best able to, this is a gross oversimplification, but a lot of times, are best able to reflect back to us what we want to hear.* —Senior-level, GN

Ultimately, participants felt the dynamic of donor-driven priorities paired with lack of partnership undermines the GS.

Participants felt the dynamic of donor-driven priorities paired with lack of partnership undermines the GS.

*Instead of trying to strengthen the health system, the funders have an alternative agenda and just want results. [The funder] succeeds but the system is left poorer and weaker…* —Senior-level, GN

Burdensome contracting processes emerged as a major hindrance to successful shifting of funds to GS for leadership of projects. One participant reflected on “prohibitive compliance requirements”^26^ irrespective of ability to implement.

*We can kill a really good organization by giving them money. . . . There should be a way to lessen some of the controls on our work so that we are not killing people by trying to help them.* —Senior-level, GN

GS-based participants also highlighted inequitable contracts between GS- and GN-based organizations, lack of power to negotiate with funders and GN organizations, lack of bandwidth to search for funding opportunities, and lack of institutional investment by funders and GS countries themselves to develop the infrastructure and capacity to administer GHD projects.

*…indirect costs for LMICs! How do they [funders]expect the organizations to flourish? They wouldn't say this to [a high-income university] … they pay [high-income universities] 3 times what they are denying an LMIC institution!* —Senior-level, GS

Participants offered numerous strategies to address these colonial legacies.

*[Solicitations should be] cocreated with beneficiaries. Sit down, talk to them, understand their problem and then you can create the solution together.* — Mid-level, GS

Other participants suggested harmonization with existing country priorities expressed in policies, agendas, and strategies. A senior-level GS participant remarked that smaller “otherwise voiceless organizations” should have a seat at the table.

To minimize administrative burdens, 1 senior-level GN participant suggested allowing GS organizations to focus on generating ideas in more innovative formats, such as videos, and suggested having a dedicated unit to assist small groups in completing paperwork. Thinking about steps GS-based organizations could take to share power and resources, a senior-level GS participant suggested forming a coalition, noting “they would significantly negotiate better as a group of countries. . . .” Thinking broadly, 1 senior-level GS participant advocated for a global framework “to define an acceptable, respectable, equitable partnership in global health.”

### Colonial Legacies in Program Planning and Implementation and Strategies to Address Them

In the planning and implementation phases of GHD programming, the unequal distribution of resources, devaluation of roles that are based in the GS, and lack of flexibility to respond to the implementation context were key themes across the challenges highlighted in interviews (Table 2).

Once funding is awarded, participants noted that contracts and partnership structures center resources and power in the GN.

*Most of the money for the grant implemented in an LMIC is actually spent in the high-income country.* —Senior-level, GS

These inequities in resource distribution were perceived to be enshrined in funder policy.

*Some of the funders… have international policies … like if they give you a fund, almost 60% of the fund will go back to USA through the researchers or the products you buy.* —Senior-level, GS

At the same time, the resources that organizations and individuals in the GS bring to GHD implementation are frequently devalued.

*So, we really need to give value to what we [in the GS] bring to the table. The research experience, the space within which the research is taking place, the expertise … the participants and all of that are essential, in fact without them, that cannot go on … The money with itself is useless without all of this and we need to appreciate that because … it is our basis for saying this is [the] … direction the research should take.* —Junior-level, GS

A mid-level GS participant stated, “local experts have access to communities and knowledge about how to best serve them.” However, participants noted that GHD imposes a rigid style of implementation and accompanying “technical assistance models,” which, they suggested, infer lack of capacity and expertise in the GS.

Participants noted that GHD imposes a rigid style of implementation that they suggested infers lack of capacity and expertise in the GS.

Participants noted how rigid, predefined results limit the impact of GHD project implementation.

*The implementation context, as you know, is not a laboratory where everything is controlled. Circumstances change, the problem evolves, but … you are limited by trying to play by the rules and you are denied flexibility and responsiveness to the context. So, if it was designed as so, it has to be delivered as so. If you have to change, then the processes have to be laborious and long …* —Junior-level, GS

To move the needle toward decolonization in program planning and implementation, participants note that funders should acknowledge the complex, dynamic context in which GHD work takes place and facilitate adaptive management by reducing burdensome processes associated with the mechanisms used to issue awards. A mid-level GN participant suggested, “build[ing] accountability and feedback practices where you are accountable to the subpartners and to the whole community.” Relevant to each phase of the cycle, 1 participant's reflection speaks to the colonial legacies raised by participants.

*… If you are really serious about decolonizing, we have to apply a power analysis to everything that we do … those of us in positions of power/authority will need to give them up and cede ground to people that we do not know.* —Senior-level, GN

### Colonial Legacies in Evaluation and Dissemination and Strategies to Address Them

When describing the program evaluation and dissemination phase, participants cited the co-option of results, data ownership issues, and outputs that are not well-suited to the local context. The themes that emerged under evaluation and dissemination and accompanying strategies to decolonize are summarized in Table 2.

Participants shared how GN partners often own the data collected by and in the GS and present the results of the work performed by the GS partner to the funder and other external audiences without acknowledging the significant contribution of the GS partner(s).

*… [the prime GN organization] will present these results [obtained by the GS partner] as if it too has made efforts, whereas sometimes it [the prime GN organization] only does monitoring and evaluation to see if the programmed activities are taking place on schedule and so on. But afterwards the organization that carries out the activities … is not presented to this funding partner as the owner of the data … We really need this equity. Until now we think that we are trampled by some so-called large organizations that do not have the experience that we have.* —Senior-level, GS

Furthermore, participants highlighted concerns around results. A senior-level GS participant noted, “the technical and financial partner is sometimes capricious and imposes its way of working and its way of harvesting results,” resonating with inflexibility described in the project implementation phase.

Participants highlighted how lack of input and partnership at the point at which outputs and goals are defined in the conceptualization phase leaves GS practitioners with less relevant evidence to disseminate in the evaluation and dissemination phase, ultimately limiting the chance to shape policy.

*… You are left in 2 different worlds – with minimal input to our policy and programming but more delivering the project deliverables. And rarely do you find that policy reforms … are 1 of the key outputs of the … research.* —Junior-level, GS

To decolonize the evaluation and dissemination phase, participants point to shared ownership of data and results, increased valuation of qualitative results for evaluation of investments, the embrace of more mutually beneficial partnerships, and shared definition of outputs to best serve local purposes. They suggest GN partners prioritize end users by sharing research and results in local languages and conveying them in accessible and relatable formats that can be used to shape policy and practice, with less focus on academic articles.

## DISCUSSION

In this article, we use qualitative methodologies to elucidate how colonial legacies are present across the GHD project cycle and identify concrete strategies to decolonize from the perspective of experienced GHD practitioners. Our findings demonstrate that when funding and decision-making power are centered in the GN at the outset, the stage is set for power imbalances that persist throughout the project life cycle. For example, even during cocreation at the design phase, power imbalances favor English-speaking GS colleagues with travel visas who can attend in-person sessions. In conceptualization and contracting, participants highlighted how priorities are set by funders and GN-based partners without building in partnership and accountability to GS partners and communities. Burdensome processes make it difficult for GS-based organizations to win and manage funds. In planning and implementation, resources are distributed inequitably, GS-based contributions are undervalued, and GN funders and partners impose expectations for implementation that preclude adaptive management. In evaluation and dissemination, participants noted how GN partners present work done by GS partners to funders as their own, GS data are owned by the GN, and outputs are not systematically designed to meet the needs of GS-based users. It is well established that these practices are rooted in and perpetuated by coloniality; they maintain power and control in the GN, while severely limiting the success and sustainability of GHD efforts.^27^ As assessed by Plamondon et al., although global health partnerships are frequently portrayed as beneficial for partners in the GS, inadequate attention has been paid to power dynamics and inequities.

Key principles underlie many of the strategies key informants proposed to decolonize across the program phases: partnership, equity, and flexibility. These key principles underscore an equity-centered approach where justice, humility, and reciprocity are central to true global health partnerships.^5^^,^^28^ GS and GN partners each have a role to play “by applying a power analysis to everything we do,” as a GN senior-level participant noted. Participants pointed to significant tangible barriers that can be removed by funders and GN partners to advance GS leadership and improve equity by de-bureaucratizing application processes and relaxing limits to allowable overhead charges. Although programs have been developed to progress in this direction and advance decolonization,^29^ it is a drop in the bucket. Participants called for less rigid approaches to implementation and results whereby they can jointly determine who plays what role and assess if the planned strategy is effective, guiding adaptation as needed. For example, participants asked for funders to pursue open communication to create more transparent, collaborative, trust-based relationships based on mutual respect between GS-funded partners, funders, and other GN organizations. Thus, funders are expected to play an active role in driving strategies to advance equity.^30^ Finally, the need for more accountability within partnerships and to communities is imperative, as GHD projects should demonstrate socioeconomic improvements in the communities they serve.^31^

Key principles underlie many of the strategies key informants proposed to decolonize across the program phases: partnership, equity, and flexibility.

Decolonizing GHD is not a quick fix; the project cycle is situated within a vast and complex system that needs to be overhauled with slow, deliberate efforts.^32^ With many donors and implementing partners based in the GN, the field of GHD will have to carefully construct a post-colonial future, transferring power and resources (both human and financial) to the GS while ceding space for GS ownership and leadership.^33^ There are numerous cross-cutting strategies, including efforts to strengthen diversity, equity, inclusion, and accessibility and movements toward localization, each addressing power imbalances with unique vantage points.^10^ These systemic strategies are broader than the project life cycle and, therefore, beyond the scope of this article but are central to decolonization efforts. We note that even the “project life cycle framework” is driven by the GN, perpetuates colonial legacies, and interferes with organic, community-led development. Although we believe GHD may “survive its decolonization,”^2^ GHD work will be of higher quality and more sustainable when resources and power are centered in the GS.

The reflection on the project life cycle presents practical next steps to shift power and embed accountability across all phases of project implementation and research as part of the journey to dismantle structural inequities in global health and development. Although this is only 1 piece of the puzzle,^34^ the call for more partnership, equity, and flexibility can be applied beyond the project cycle. We note that GS partners need to invest in building systems that can sustain effective, accountable, and equitable leadership for GHD. This will require continuous reflection for “self-decolonization” and decolonization of global health and development, acknowledging that, in several instances, GS GHD researchers and practitioners have played a role in “perpetuating coloniality” when working with the “local Global South,” such as community partners and vulnerable populations.”^35^^,^^36^ Ultimately, our work reinforces existing calls to recenter GS expertise, leadership, knowledge, and solutions in all phases of GHD work. Resources, both financial and human, must be allocated to support this seismic shift.^7^^,^^37^^,^^38^

### Limitations

Our approach is subject to limitations. Results are not designed to be generalized or to make quantitative comparisons between participant type, organization, or region. Although we pursued diversity in participant profiles, key informants were highly educated and possessed deep knowledge about the issues of decolonization. Thematic saturation was achieved quickly, which may speak to the nature of key informant interviews and again limits generalizability. Because we selected key informants from our professional networks, we may not have comprehensively captured views from networks of community-based organizations. Although worth noting, we believe that this positionality did not bias our interpretation of the results because of the strengths of our approach. Strengths included the fact that decision-making authority rested with GS colleagues, represented by lead authorship, and ongoing conversations for interpretation of findings accounted for our unique identities. We worked on this article in a highly collaborative way, which enabled validation and quality check because reflexivity, interpretation of findings, and clarification of meaning occurred throughout the entire research process (including French translation of materials). Future research should include more diversity of respondents, though our informants did include junior colleagues and a majority of GS-based individuals. Despite these limitations, our study contributes tangible steps to move from ideas to action, identifying concrete examples of power imbalances in GHD's current iteration while offering accompanying strategies to decolonize.

## CONCLUSIONS

Given the persistence of inequities throughout the project life cycle and the availability of actionable, project phase-based strategies to address GH's power asymmetry, all GHD partners, including individual practitioners, community organizations, nongovernmental organizations, and funders, among others, must commit to transform work across the program cycle to redistribute power and resources, which will ultimately enhance the value, impact, and sustainability of GHD projects. We do not advocate for a complete disassembly of GHD, but instead, we invite critical reflection; respectful, intentional relationships; the recentering of GS expertise; and the transfer of power and resources from GN to GS in GHD. Standards should be put in place with metrics used to monitor and track this process and to assess its impact. Future research should explore these components with emphasis on how to systematically transfer power and resources to the GS, which will ultimately advance decolonization.

## Supplementary Material

GHSP-D-23-00187-supplement.pdf

## Notes

**Peer Reviewed**

**First published online:** October 26, 2023.
