# Supplementary material for: Applying a Power Analysis to Everything We Do: A Qualitative Inquiry to Decolonize the Global Health and Development Project Cycle
Source: Glob Health Sci Pract. 2023 Oct 30;11(5):e2300187. doi: 10.9745/GHSP-D-23-00187 (PMC10615245; doi:10.9745/GHSP-D-23-00187)
Supplement: GHSP-D-23-00187-supplement.pdf [file GHSP-D-23-00187-supplement.pdf]

**CODEBOOK**	
**Codes**	**Code definition**
Code Family: **Project Life Cycle**	This code family organizes the sequential stages of the inception of a project (implementation or research), distribution of funds from the Global North to Global South (in the context of this paper) all through implementation of the project, evaluation and dissemination<br>It also applies to how local partners engage with each other e.g., a Global South Partner collaborating with a local NGO or local health facility to conceptualize and implement a service delivery project or research study in the community<br><br>NOTE: Each phase of the project life cycle is coded as either a legacy of colonialism (examples of colonial practices that propagate imbalance and inequity) or a strategy to decolonize (tangible operational recommendations to decolonize processes and partnerships to achieve balance, equity and localization) While the terms legacy of colonialism/ strategies to decolonize do not fit the data perfectly in every instance, these can be used to generally organize the data into negative experiences across the project cycle or positive aspirations to achieve equitable partnerships all levels (for ALL partnerships not just Global North and Global South).
Conceptualization_Legacy of Colonialism	This code represents experiences of neocolonialism in the earliest phase of a project or partnership formation. Includes proposal design driven by funders, early thinking about what "problem" the "funding resources" will address, how background research is conducted and how priorities are set.
Conceptualization_Strategies to Decolonize	This code represents efforts to reform the earliest phase of "funding resources" to be repsonsive to the movement to decolonize processes and decisions. Includes strategies to overcome related to proposal design by funders, early thinking about what "problem" "funding resources" will address, how background research is conducted and how priorities are set

Planning_Legacy of Colonialism	This code represents experiences of neocolonialism in contract negotiation, business development, partnership planning, activity planning, protocol development, defining outcomes and initial stakeholder engagments (who is on the table and whose voices count in deciding on the plan of action)
Planning_Strategies to Decolonize	This code represents efforts to reform the planning of "funding resources" to be repsonsive to the movement to decolonize, including contract negotiation, business development, partership planning, activity planning, protocol, defining outcomes and initial stakeholder engagements (who is on the table and whose voices count in deciding on the plan of action)
Implementation_Legacy of Colonialism	This code represents experiences of neocolonialism in project or research implementation, data collection, community outreach and monitoring of outcomes or results
Implementation_Strategies to Decolonize	This code represents efforts to reform the implemetation of project activities to be repsonsive to the movement to decolonize, including project or research implementation, data collection, community outreach and monitoring of outcomes or results.
Evaluation_Legacy of Colonialism	This code represents experiences of neocolonialism in evaluation of project activities and measurement of results including indicators used to assess success and undervaluing community engagement and entry
Evaluation_Strategies to Decolonize	This code represents efforts to reform the implemetation and evaluation of project activities to be repsonsive to the movement to decolonize evaluation processes, measurements and indicators to promote holistic evaluation of project activities and valuing community engagement and entry activities
Dissemination and Reporting_Legacy of Colonialism	This code represents experiences of neocolonialism in the way results are reported and shared, as well as to whom and by whom.
Dissemination and Reporting_Strategies to Decolonize	This code represents efforts to reform the disseminatoin and reporting of results, including to whom and by whom, in ways that are repsonsive to the movement to decolonize knowledge and achieve shared ownership of acheievements and challenges
